# Risk Factors for Severe Cholera among Children under Five in Rural and Urban Bangladesh, 2000–2008: A Hospital-Based Surveillance Study

**DOI:** 10.1371/journal.pone.0054395

**Published:** 2013-01-18

**Authors:** Danny V. Colombara, Karen D. Cowgill, Abu S. G. Faruque

**Affiliations:** 1 Department of Epidemiology, University of Washington, Seattle, Washington, United States of America; 2 Department of Global Health, University of Washington, Seattle, Washington, United States of America; 3 Seattle University College of Nursing, Seattle, Washington, United States of America; 4 International Centre for Diarrhoeal Disease Research, Bangladesh, Dhaka, Bangladesh; The Australian National University, Australia

## Abstract

**Background:**

Children under five bear the largest cholera burden. We therefore sought to identify modifiable risk factors among Bangladeshi children.

**Methodology/Principal Findings:**

We used multivariate Poisson regression to assess risk factors for severe cholera among diarrheal patients presenting at hospitals in Matlab (rural) and Dhaka (urban), Bangladesh. Risk increased with age. Compared to those under one, rural and urban four-year-olds had adjusted risk ratios (aRR) of 4.17 (95% confidence interval (CI) 2.43–7.15) and 6.32 (95% CI: 4.63–8.63), respectively. Breastfeeding halved the risk in both rural (aRR = 0.49, 95% CI: 0.35–0.67) and urban (aRR = 0.51, 95% CI: 0.41–0.62) settings. Rural children’s risk decreased with maternal education (P-trend: <0.001) and increased among those with a family member with diarrhea in the past week (aRR = 1.61, 95% CI: 1.22–2.14) and those with prior vitamin A supplementation (aRR = 1.65, 95% CI: 1.12–2.43). Urban children whose mothers daily (aRR = 0.41, 95% CI: 0.21–0.79) or occasionally (aRR = 0.55, 95% CI: 0.36–0.84) read a newspaper experienced reduced risk. Urban children from households with incomes between 34–84 USD/month had a 30% increased risk compared to those from households with incomes >84 USD/month.

**Conclusion/Significance:**

Increasing age, lower socioeconomic status, and lack of breastfeeding are key correlates of increased risk for cholera hospitalization among those under five in rural and urban Bangladesh. In addition, having a family member with diarrhea in the past week was associated with increased risk among rural children. Continued attention should be directed to the promotion of breastfeeding. Further research is needed to elucidate the relationship between maternal education and cholera risk. Renewed research regarding the use of chemoprophylaxis among family members of cholera cases may be warranted in rural endemic settings.

## Introduction

Cholera is a potentially life-threatening, primarily waterborne, diarrheal disease caused by infection with *Vibrio cholerae* bacteria. A 2012 review of cholera’s global burden estimated that 1.4 billion people are at risk for cholera, with 2.8 million cases and 91,000 related deaths in endemic regions annually [1]. This burden is disproportionately borne by the young, with children under five having the highest incidence of cholera and contributing almost half of the mortality [1]. More than 40 years ago it was reported that the cholera case fatality rate among children one to five years old was more than 10 times that of adults [2], but description of this disparity has not resulted in large-scale studies of cholera risk factors unique to young children. Most prior studies have been small [3], focused on specific risk factors such as breastfeeding [4], [5], or assessed risk factors for diarrhea in general [6]. More recently, other studies have examined risks for duration of diarrheal illness [7] and diarrheal disease associated death [8], [9]. Research that specifically explores cholera risk factors in children under five may provide an important new perspective.

Although an oral bivalent cholera vaccine campaign was initiated in response to the epidemic that started in Haiti in 2010, and a large-scale feasibility trial of this same vaccine began in Bangladesh in 2011 (clinicaltrials.gov id: NCT01339845), there is currently no evidence that this vaccine will be able to stop an epidemic or significantly reduce cholera burden in endemic settings. Furthermore, a 2011 Cochrane review reported that the protective efficacy of five types of killed whole cell cholera vaccine in children under five was significantly lower than among older children and adults [10].

Due to uncertainty regarding the efficacy of cholera vaccines in young children, we sought to identify risk factors amenable to non-immunologic intervention by developing predictive models for severe cholera in children under five. Due to substantial environmental and socioeconomic regional differences, we examined children in rural (Matlab) and urban (Dhaka) Bangladesh separately.

## Methods

### Ethics Statement

Hospital surveillance activities were approved by the Ethical Review Committee (ERC) and the Research Review Committee (RRC) of the International Centre for Diarrhoeal Disease Research, Bangladesh (ICDDR,B). Informed oral consent was obtained from all participants and was documented in the DDSS database by ICDDR,B staff. For minors, informed oral consent was obtained from parents, guardians, caretakers, or next of kin. Anonymized medical records were used in all data analyses. This research was exempted from human subjects review by the University of Washington Institutional Review Board. The ERC and RRC approved the use of oral consent because of the high proportion of illiterate patients.

### Study Design & Setting

We performed a hospital-based surveillance study using the Diarrhoeal Diseases Surveillance System (DDSS) databases from Matlab and Dhaka ICDDR,B hospitals. The DDSS records clinical, demographic, socioeconomic, and enteric pathogen data from diarrheal patients. All DDSS patients had stool cultured for enteric pathogens following standard bacteriological methods [11]–[13]. In addition to *V. cholerae*, stools were systematically tested for rotavirus, *Shigella*, *Salmonella,* amoeba and *Giardia* species.

Matlab Upazila (sub-district) is a predominantly rural area of Bangladesh, with villagers comprising more than 97% of the population. In Matlab, a Health and Demographic Surveillance System (HDSS) covering approximately 200,000 residents was established in 1966. All diarrheal patients living in the HDSS catchment area were enrolled in the DDSS.

Dhaka is a densely populated city, with more than 12 million residents in 2008. Every fiftieth diarrheal patient visiting the Dhaka hospital has been enrolled in the DDSS since 1996 [12]. In both settings, ICDDR,B hospitals provide excellent diarrhea treatment at no cost to the patient.

For this analysis, Matlab patients who lived in villages were considered rural dwellers. Dhaka patients who lived in high-density residential or mixed-use areas, or slums, were considered urban dwellers.

### Study Population

We limited the analysis to patients under five years old entering ICDDR,B hospitals between January 1, 2000 and December 31, 2008. Patients missing age data, Dhaka patients residing in villages, and Matlab patients who reported residing in slums or high-density residential or mixed use areas were excluded. Due to the inability to attribute severe diarrhea to a particular pathogen in patients who were co-infected with *V. cholerae* and another pathogen, we excluded these patients. There were no other exclusion criteria. Multiple hospital admissions of the same child could not be identified because anonymized data were used.

### Cholera Definition

Laboratory-confirmed culture of *V. cholerae* (negative, positive) was the outcome of interest. *V. cholerae* status was positive when *V. cholerae* O1 Classical Inaba, *V. cholerae* O1 Classical Ogawa, *V. cholerae* O1 El Tor Inaba, *V. cholerae* O1 El Tor Ogawa, or *V. cholerae* O139 (Bengal) was recorded as one of the first three isolated pathogens in the DDSS database.

### Data Analysis

Characteristics of cholera and non-cholera patients, stratified by rural or urban residence, were compared using chi-squared tests for categorical variables. The Mann–Whitney U test was used for the number of household members, a continuous variable with a right-skewed distribution. Sociodemographic characteristics were self-reported and included age, sex, the number of household members, maternal education, maternal newspaper readership, monthly household income, residence in a slum, home ownership, and the presence of concrete floors in the home.

Self-reported water and sanitation characteristics included household toilet facilities, distance from the kitchen to drinking water, sources of drinking and washing/bathing water, and drinking water treatment.

Other variables of interest included a family member with diarrhea in the past week, breastfeeding status, history of vitamin A supplementation, and distance from home to the hospital, all of which were self-reported. Severe acute malnutrition (mid-upper arm circumference <11.5 cm) was assessed by ICDDR,B medical personnel.

Clinical characteristics included the general condition on admission (assessed by medical personnel), diarrhea duration prior to arrival (self-reported), degree of clinical dehydration (assessed by medical personnel), watery stool, stool contents, and the number of stools and bouts of vomiting in the 24 hours prior to admission (all self-reported). We also assessed the distribution of enteric pathogens detected by ICDDR,B laboratories.

### Risk Factors Examined

Sociodemographic risk factors examined were age, sex, maternal education (none, 1–5, 6–9, 10–12, >12 years), maternal newspaper readership (never, <7 days/week, daily), monthly household income (84+, 50–84, 34–50, <34 USD (converted from Taka at 59.4 Taka/USD, the mid-market rate on July 1, 2004, the mid-point of our study [14])), residence in a slum (yes, no), home ownership (own, rent), and concrete floors in the home (yes, no).

Water and sanitation risk factors included household toilet facilities (improved or unimproved, as defined by the WHO/UNICEF Joint Monitoring Programme for Water Supply and Sanitation [15]), distance from the kitchen to drinking water (10-meter increments), water source (ranked in decreasing order of safety as tap, tube well, or surface; if different sources were used for drinking and washing/bathing water, the least safe source was analyzed), and drinking water treatment (none, boiling, other). Water sources used in food preparation were not available. Surface water was defined as water from a pond, river, or ditch. Other water treatment included filtering, sieving, and treatment with tablets.

A history of a family member with diarrhea in the past week (yes, no), current breastfeeding (yes, no), severe acute malnutrition (yes, no), prior vitamin A supplementation (never, ever), and distance from home to the hospital (5 km increments) were also assessed.

### Statistical Methods

Risk ratios (RR) and 95% confidence intervals (95% CI) for cholera risk factors were assessed using Poisson regression, with robust variance estimates to compensate for variance overestimation [16]. Candidate risk factors with more than 5% missing data were excluded from analysis. A linear trend test was performed for ordinal variables with ≥4 strata.

Univariate risk factors associated with cholera with a p-value <0.10 and an RR of <0.9 or >1.1 were candidates for the multivariate model. We excluded risk factors with an RR between 0.9 and 1.1 because of the likelihood that weak (epidemiologically unimportant) associations would be found statistically significant solely due to our large sample size. Collinearity among multivariate candidates was assessed using variance inflation factors (VIF), with a VIF of ≥10 indicating collinearity. If collinear candidates were found, only the predictor judged to be more biologically plausible was considered for the multivariate model.

A multivariate regression model was built by adding and testing candidate predictors individually, in order of effect size. Continuous predictors were retained if the Wald test was significant (p-value <0.05). Retention of categorical predictors was also dependent on a significant Wald test for at least one stratum. Since regression with robust standard errors does not provide log likelihoods, we could not perform likelihood ratio tests to compare models. After inclusion in the model, risk factors were not reevaluated in subsequent model building steps.

Sex, the number of household members (continuous), and cholera seasonality were *a priori* confounders and were included in the multivariate model as adjustment variables. Seasonality was constructed by creating a restricted cubic spline of the day of the year on the date of visit (1–366) with seven knots [17].

Analyses used two-sided significance levels and were performed with Stata/IC 11.2 (StataCorp LP, College Station, TX).

## Results

### Study Population

**Figure 1:**
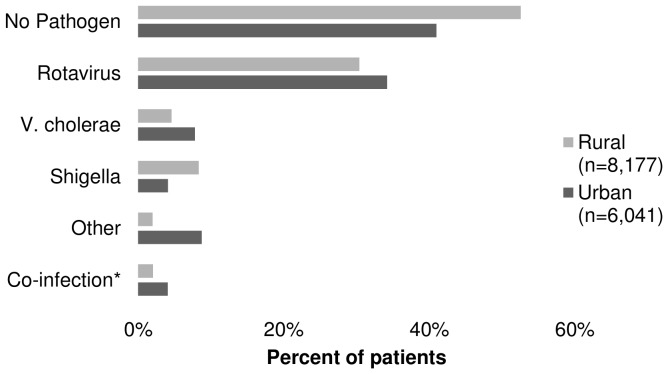
Frequency of detected enteric pathogens among children under five in ICDDR,B hospitals, Bangladesh, 2000–2008. * Co-infection is defined as a positive assay for two or more non-cholera pathogens. Patients with *V. cholerae* co-infection were excluded from analyses.

Of the 13,839 Matlab patient records in the DDSS, four (<0.01%) had missing age data, 304 (2.2%) were non-rural dwellers, 5,193 (37.5%) were older than five, and 161 (1.2%) had laboratory-confirmed co-infections with *V. cholerae* and another enteric pathogen. Among the remaining 8,177 rural children, there were 378 (4.6%) laboratory-confirmed *V. cholerae* cases. Of the 19,332 Dhaka patient records, 41 (0.2%) had missing age data, 6,938 (36%) were non-urban dwellers, 6,176 (31.9%) were older than five, and 136 (0.7%) had laboratory-confirmed infections with *V. cholerae and another enteric pathogen.* Among the remaining 6,041 urban children, there were 473 (7.8%) cases of cholera. Dhaka had a higher proportion of cholera cases (*P*<0.001). A known pathogen was detected in 47% of rural patients and 59% of urban patients (Figure 1).

### Comparison of Cholera and Non-cholera Diarrhea Cases

In the rural setting, cholera patients were older (median age 30 vs. 14 months), had fewer household members, and were more likely to have uneducated mothers who never read newspapers than other diarrhea patients (Table 1). They also came from families with lower household incomes and their homes were less likely to have concrete floors, improved toilet facilities (6.3% vs. 10.2%), and a drinking water source within 10 meters of their kitchen (41.5% vs. 55.9%). They were more likely to use surface water for drinking or washing, to have had a family member with diarrhea in the past week (11.6% vs. 6.2%), and less likely to be breastfed (48.8% vs. 88.4%) and have severe acute malnutrition. They were also more likely to have had prior vitamin A supplementation (82.3% vs. 49.9%) and to live further from the hospital than other diarrhea patients.

**Table 1:** Sociodemographic, water and sanitation, and other potential correlates of diarrhea among diarrheal patients <5-years-old in ICDDR,B hospitals, Bangladesh, 2000–2008.

	Rural													Urban				
	Cholera (n = 378)						Other (n = 7,799)							Cholera (n = 473)		Other (n = 5,568)		
	N			%			N			%			*P* a	N	%	N	%	*P* a
**Sociodemographic**																		
** Age (yr)**													<0.001					<0.001
0	65			17.2			4056			52.0				103	21.8	3397	61.0	
1	75			19.8			2628			33.7				112	23.7	1496	26.9	
2	108			28.6			651			8.3				102	21.6	358	6.4	
3	74			19.6			286			3.7				88	18.6	198	3.6	
4	56			14.8			178			2.3				68	14.4	119	2.1	
** Female sex**	154			40.7			3043			39.0			0.503	215	45.5	2252	40.4	0.033
** No. household members** b	5			2.1			5			2.4			0.010	4	1.9	4	2.3	0.751
** Maternal education (yr)**													<0.001					<0.001
None	142			37.6			1659			21.3				216	45.7	1842	33.1	
1–5	99			26.2			1983			25.4				105	22.2	1138	20.4	
6–9	110			29.1			2974			38.1				106	22.4	1410	25.3	
10–12	25			6.6			922			11.8				27	5.7	721	12.9	
>12	2			0.5			261			3.3				19	4.0	457	8.2	
** Maternal newspaper readership**													0.004					<0.001
Never	347			92.0			6756			86.8				440	93.4	4662	84.1	
<7 days/week	29			7.7			855			11.0				22	4.7	568	10.2	
Daily	1			0.3			175			2.2				9	1.9	315	5.7	
** Monthly household income (USD)**													<0.001					<0.001
84+	142			37.6			3850			49.4				188	39.7	2889	51.9	
50–84	169			44.7			3091			39.6				210	44.4	1965	35.3	
34–50	57			15.1			774			9.9				62	13.1	543	9.8	
<33	10			2.6			84			1.1				13	2.7	171	3.1	
** Residence in a slum**	–			–			–			–				101	21.4	827	14.9	<0.001
** Homeowner**	367			97.1			7558			96.9			0.854	49	10.4	823	14.8	0.009
** Concrete floor in home**	24			6.3			911			11.7			0.001	342	72.3	4405	79.1	0.001
**Water & Sanitation**																		
** Improved toilet facilities**	24			6.3			792			10.2			0.016	285	60.3	3977	71.4	<0.001
**Distance to drinking water (10 m increments)**											<0.001							<0.001
0		0			0		20			0.3				79	16.7	1661	29.8	
<1		157			41.5		4332			55.6				282	59.6	2860	51.4	
1–2		58			15.3		1229			15.8				79	16.7	689	12.4	
2–5		71			18.8		1146			14.7				25	5.3	295	5.3	
5+		92			24.3		1071			13.7				8	1.7	62	1.1	
** Water source**												0.001						0.035
Tap			0		0		46		0.6					392	82.9	4843	87.0	
Tube well			37		9.8		1280		16.4					77	16.3	678	12.2	
Surface^c^			341		90.2		6467		83.0					4	0.8	44	0.8	
** Drinking water treatment**												0.099						<0.001
None			346		91.5		7334		94					291	61.5	2658	47.7	
Boiling			7		1.9		127		1.6					179	37.8	2851	51.2	
Other			25		6.6		337		4.3					3	0.6	58	1.0	0.069
**Other Potential Correlates**																		
** Family member with diarrhea in past week**			44		11.6		484		6.2			<0.001		76	16.1	560	10.1	<0.001
** Currently breastfed**			182		48.4		6878		88.4			<0.001		235	50.0	4574	82.3	<0.001
** Severe acute malnutrition** d			19		5.0		624		8.0			0.035		63	14.1	986	18.1	0.032
** Prior vitamin A supplementation**			311		82.3		3891		49.9			<0.001		252	53.3	2659	47.8	0.021
** Distance to hospital (km)**												0.004						<0.001
≤3			61		16.1		1782		22.8					19	4.0	451	8.1	
>3 & ≤5			122		32.3		2562		32.9					18	3.8	373	6.7	
>5 & ≤7			55		14.6		1112		14.3					39	8.2	628	11.3	
>7			140		37.0		2343		30.0					397	83.9	4115	73.9	

a Two-sided chi-squared test for categorical variables and Mann-Whitney U test for continuous variables; cholera vs. other diarrhea.

b Median and standard deviation provided for continuous variables.

c Surface water includes ponds, rivers, and ditches.

d Severe acute malnutrition is defined as mid-upper arm circumference (MUAC) <11.5 cm.

Similar relationships were observed for cholera vs. other diarrhea patients in the urban setting, with the following exceptions. Urban cholera patients were more likely to be female (45.5% vs. 40.4%) and did not differ significantly in the number of household members compared to non-cholera patients. Cholera patients were also more likely to reside in a slum (21.4% vs. 14.9%) and less likely to come from a family that owned a home, to use tap water, or to treat their drinking water.

In both settings, cholera patients were less likely to present in normal physical condition, and more likely to present within one day of diarrhea onset than other diarrhea patients (Table 2). They were also more likely to have severe dehydration, watery stool, non-bloody, non-mucusy stool contents, and more than 10 bowel movements and vomiting in the prior 24 hours.

**Table 2:** Clinical characteristics of diarrheal patients <5-years-old in ICDDR,B hospitals, Bangladesh, 2000–2008.

	Rural								Urban					
	Cholera (n = 378)		Other (n = 7,799)						Cholera (n = 473)		Other (n = 5,568)			
	N	%	N	%				*P* ^a^	N	%	N	%		*P* ^a^
**Clinical Characteristics**														
** General Condition**								<0.001						<0.001
Normal	222	58.7	7026	90.2					85	18.0	3552	63.9		
Restless	72	19.0	542	7.0					6	1.3	48	0.9		
Lethargic but irritable	73	19.3	207	2.7					237	50.1	1828	32.9		
Drowsy/cold & sweating	11	2.9	12	0.2					145	30.7	132	2.4		
** Duration of diarrhea prior to arrival (days)**								<0.001						<0.001
<1	186	49.2	2430	31.2					223	47.1	1237	22.2		
1–6	182	48.1	5045	64.7					229	48.4	3818	68.6		
7–14	9	2.4	280	3.6					18	3.8	434	7.8		
15+	1	0.3	44	0.6					3	0.6	78	1.4		
** Clinical Dehydration**								<0.001						<0.001
None	154	40.7	6705	86.0					84	17.8	3549	63.8		
Some	164	43.4	1052	13.5					229	48.4	1865	33.5		
Severe	60	15.9	39	0.5					160	33.8	146	2.6		
** Watery stool**	320	84.7	5641	72.3				<0.001	466	98.5	5239	94.1		<0.001
** Stool contents**								<0.001						<0.001
Normal	314	83.1	5057	64.8					419	88.6	4335	77.9		
Mucus	42	11.1	1657	21.2					51	10.8	1077	19.3		
Blood	3	0.8	106	1.4					1	0.2	6	0.1		
Mucus + Blood	19	5.0	979	12.6					2	0.4	150	2.7		
** No. stools in 24 hours prior to arrival**								0.001						<0.001
3–5	38	10.1	792	10.2					39	8.2	501	9.0		
6–10	187	49.5	4415	56.6					222	46.9	3225	57.9		
11–14	81	21.4	1680	21.5					150	31.7	1199	21.5		
15–20	46	12.2	565	7.2					36	7.6	400	7.2		
21+	26	6.9	347	4.4					26	5.5	243	4.4		
** Vomiting in 24 Hours prior to arrival**						<0.001								<0.001
None	54	14.3	2604	33.4					39	8.2	1103	19.8		
<10 times	256	67.7	4653	59.7					387	81.8	4249	76.3		
10+ times	68	18.0	542	6.9					47	9.9	216	3.9		

### Risk Factor Analysis

Rural and urban univariate analysis results are reported in Tables 3 and 4, respectively. The data for all risk factors were considered complete (≤5% missing), and we found no instances of collinearity among the assessed variables.

**Table 3:** Assessment of risk factors for severe cholera among rural children <5-years-old in Matlab, Bangladesh, 2000–2008.

		Univariate				Multivariate^a^		
		(n = 8,177)				(n = 8,159)		
		Cholera/Total (%)		RR	95% CI	Cholera/Total (%)	RR	95% CI
**Sociodemographic**								
** Age (yr)** b								
0		65/4121 (2)		1		65/4113 (2)	1	
1		75/2703 (3)		1.76	(1.27–2.44)	75/2698 (3)	1.17	(0.77–1.78)
2		108/759 (14)		9.02	(6.70–12.20)	108/758 (14)	3.84	(2.42–6.07)
3		74/360 (21)		13.03	(9.51–17.90)	74/358 (21)	3.91	(2.32–6.58)
4		56/234 (24)		15.17	(10.88–21.2)	54/232 (23)	4.17	(2.43–7.15)
** Female sex**		154/3197 (5)		1.07	(0.88–1.31)			
** Mother’s education (yr)** c								
None		142/1801 (8)		1		142/1799 (8)	1	
1–5		99/2082 (5)		0.60	(0.47–0.77)	98/2076 (5)	0.70	(0.55–0.88)
6–9		110/3084 (4)		0.45	(0.36–0.58)	109/3078 (4)	0.61	(0.48–0.77)
10–12		25/947 (3)		0.33	(0.22–0.51)	25/945 (3)	0.45	(0.30–0.68)
>12		2/263 (1)		0.10	(0.02–0.39)	2/261 (1)	0.13	(0.03–0.52)
** Maternal newspaper readership**								
Never		347/7103 (5)		1				
<7 days/week		29/884 (3)		0.67	(0.46–0.98)			
Daily		1/176 (1)		0.12	(0.02–0.82)			
** Monthly household income (USD)**								
84+		142/3992 (4)		1				
50–84		169/3260 (5)		1.46	(1.17–1.81)			
34–50		57/831 (7)		1.93	(1.43–2.60)			
<33		10/94 (11)		2.99	(1.63–5.49)			
** Homeowner**		367/7925 (5)		1.06	(0.59–1.90)			
** Concrete floors in home**		24/935 (3)		0.52	(0.35–0.79)			
**Water & Sanitation**								
** Improved toilet facilities**		24/816 (3)		0.61	(0.41–0.92)			
** Distance to drinking water** d		–		1.03	(1.02–1.05)			
** Water source**								
Tap^e^		0/46 (0)		–	–			
Tube well		30/1317 (3)		1				
Surface^f^		341/6808 (5)		1.78	(1.28–2.49)			
** Drinking water treatment**								
None	346/7680 (5)		1					
Boiling	7/134 (5)		1.16		(0.56–2.40)			
Other	25/362 (7)		1.53		(1.04–2.27)			
**Other Potential Risk Factors**								
** Family member with diarrhea in past week**	44/528 (8)		1.91		(1.41–2.58)	44/527 (8)	1.61	(1.22–2.14)
** Currently breastfed**	182/7060 (3)		0.15		(0.12–0.18)	182/7060 (3)	0.49	(0.35–0.67)
** Severe acute malnutrition** g	19/643 (3)		0.62		(0.39–0.97)			
** Prior vitamin A supplementation**	311/4202 (7)		4.39		(3.38–5.70)	309/4194 (7)	1.65	(1.12–2.43)
** Distance to hospital** h	–		1.07		(1.02–1.12)			

a Adjusted for sex, the number of household members, seasonality, and the other predictors in the model. The number of patients in the multivariate analysis was less than the number in the univariate analysis due to missing breast feeding data for 18 children.

b Linear trend for increasing risk with age in the univariate and multivariate models (P<0.001).

c Linear trend for decreasing risk with increasing maternal education in the univariate and multivariate models (P<0.001).

d Per 10 meter increment.

e Too few observations to develop a risk estimate for rural children.

f Surface water includes ponds, rivers, and ditches.

g Severe acute malnutrition defined as mid-upper arm circumference (MUAC) <11.5cm.

h Per five kilometer increment.

**Table 4:** Assessment of risk factors for severe cholera among urban children <5–years-old in Dhaka, Bangladesh, 2000–2008.

		Univariate			Multivariate^a^		
		(n = 6,041)			(n = 6,008)		
		Cholera/Total (%)	RR	95% CI	Cholera/Total (%)	RR	95% CI
**Sociodemographic**							
** Age (yr)** b							
0		103/3500 (3)	1		101/3476 (3)	1	
1		112/1608 (7)	2.37	(1.82–3.07)	111/1603 (7)	2.33	(1.80–3.02)
2		102/460 (22)	7.53	(5.83–9.73)	102/459 (22)	5.53	(4.26–7.19)
3		88/286 (31)	10.46	(8.08–13.5)	87/284 (31)	5.86	(4.38–7.83)
4		68/187 (36)	12.36	(9.45–16.2)	68/186 (37)	6.32	(4.63–8.63)
** Female sex**		215/2467 (9)	1.21	(1.01–1.44)			
** Mother’s education (yr)**							
None		216/2058 (10)	1				
1–5		105/1243 (8)	0.80	(0.64–1.01)			
6–9		106/1516 (7)	0.67	(0.53–0.83)			
10–12		27/748 (4)	0.34	(0.23–0.51)			
>12		19/476 (4)	0.38	(0.24–0.60)			
** Maternal newspaper readership**							
Never		440/5102 (9)	1		438/5094 (9)	1	
<7 days/week		22/590 (4)	0.43	(0.28–0.66)	22/590 (4)	0.55	(0.36–0.84)
Daily		9/324 (3)	0.32	(0.17–0.62)	9/324 (3)	0.41	(0.21–0.79)
** Monthly household income (USD)**							
84+		188/3077 (6)	1		186/3062 (6)	1	
50–84		210/2175 (10)	1.58	(1.31–1.91)	208/2161 (10)	1.33	(1.10–1.61)
34–50		62/605 (10)	1.68	(1.28–2.20)	62/602 (10)	1.34	(1.03–1.75)
<33		13/184 (7)	1.16	(0.67–1.99)	13/183 (7)	1.05	(0.63–1.73)
** Residence in a slum**		101/928 (11)	1.50	(1.21–1.84)			
** Homeowner**		49/872 (6)	0.69	(0.52–0.91)			
** Concrete floors in home**		342/4747 (7)	0.71	(0.59–0.86)			
**Water & Sanitation**							
** Improved toilet facilities**		285/4262 (7)	0.63	(0.53–0.75)			
** Distance to drinking water** c		–	1.04	(1.01–1.06)			
** Water source**							
Tap		392/5235 (7)	0.73	(0.58–0.93)			
Tube well		77/755 (10)	1				
Surface^d^		4/48 (8)	0.82	(0.31–2.14)			
** Drinking water treatment**							
None	291/2949 (10)		1				
Boiling	179/3030 (6)		0.60	(0.50–0.72)			
Other	3/61 (5)		0.50	(0.16–1.51)			
**Other Potential Risk Factors**							
** Family member with diarrhea in past** **week**	76/636 (12)		1.63	(1.29–2.05)			
** Currently breastfed**	235/4809 (5)		0.25	(0.21–0.30)	235/4808 (5)	0.51	(0.41–0.62)
** Severe acute malnutrition** e	63/1049 (6)		0.76	(0.58–0.98)			
** Prior Vitamin A supplementation**	252/2911 (9)		1.23	(1.03–1.46)			
** Distance to hospital** f	–		1.01	(0.99–1.03)			

a Adjusted for sex, the number of household members, seasonality, and the other predictors in the model. The number of patients in the multivariate analysis was less than the number in the univariate analysis due to missing breast feeding (n = 12) and maternal newspaper readership (n = 21) data for urban children.

b Linear trend for increasing risk with age in the univariate and multivariate models (P<0.001).

c Per 10 meter increment.

d Surface water includes ponds, rivers, and ditches.

e Severe acute malnutrition defined as mid-upper arm circumference (MUAC) <11.5cm.

f Per five kilometer increment.

In the rural model, cholera risk increased with age (*P*-trend: <0.001) and decreased monotonically with higher levels of maternal education (*P-*trend: <0.001), when adjusted for *a priori* confounders and the other predictors in the final model (Table 3). Four-year-olds faced more than four times the risk of those less than one (adjusted risk ratio (aRR) = 4.17, 95% CI: 2.43–7.15). There was an 87% risk reduction for those with more than 12 years of education (aRR = 0.13, 95%CI: 0.03–0.52), compared to those with no formal education. Having a family member with diarrhea in the past week was associated with increased risk (aRR = 1.61, 95% CI: 1.22–2.14), whereas current breastfeeding (aRR = 0.49, 95% CI: 0.35–0.67) halved the risk. Prior vitamin A supplementation (aRR = 1.65, 95% CI: 1.12–2.43) was associated with increased risk for severe cholera.

In the urban multivariate model, cholera risk also increased with age (*P*–trend: <0.001) and was halved with current breastfeeding (aRR = 0.51, 95% CI: 0.41–0.62) (Table 4). Daily (aRR = 0.41, 95% CI: 0.21–0.79) and occasional (aRR = 0.55, 95% CI: 0.36–0.84) maternal newspaper readership were associated with reduced risk, compared to children whose mothers did not read newspapers. Children from households with incomes between 34 and 84 USD per month experienced a 30% increased risk, compared to those from households with monthly incomes greater than 84 USD.

## Discussion

Several factors emerged from this analysis that differentiated children with cholera from those with other types of diarrhea. Some findings were common to both the urban and rural setting, while others were limited to one setting or the other. Increasing age was strongly associated with cholera risk in both urban and rural settings, with a four-fold increased risk for rural four-year-olds and a six-fold increased risk for urban four-year-olds compared to those under one. Current breastfeeding, a behavior that can be successfully promoted [18], halved the risk in both settings. Socioeconomic status (SES) indicators were also key correlates of cholera risk in both settings: increasing maternal education was associated with decreasing cholera risk in rural children, and maternal newspaper readership and increasing family income was associated with decreased risk in urban children.

In the rural setting, children with a history of vitamin A supplementation or a family member with diarrhea had increased risk.

Cholera hospitalization risk increased after age two among rural children and after one among urban children. This finding is similar to that reported in a 1982 study in which rural children under two experienced hospitalization for cholera less frequently than those two to nine years old [19]. The delayed onset of risk among rural children may be explained, in part, by the greater proportion of rural mothers who breastfed their children. In addition, although we did not have data to assess this, women in rural settings may breastfeed longer than those in urban settings [20]. Early weaning may increase cholera risk through loss of cholera-specific IgA antibodies, which can be passed through breast milk and effectively protect against cholera disease in children who are colonized [21]. The protective effect of breastfeeding maybe especially pronounced in this dataset because breastfeeding does not appear to protect against rotavirus infection [22], which accounted for approximately one third of the other diarrhea in the DDSS.

In both settings, measures of maternal education – years of schooling or newspaper readership – were more strongly associated with reduced cholera risk than breastfeeding; similar findings were reported more than 35 years ago [23]. While the mechanism by which maternal education reduces cholera risk has not been specifically described, this finding underscores the importance of working toward Millennium Development Goal #2 (to achieve universal primary education) not only as a goal in its own right but also as a strategy to reduce child mortality (MDG #4).

The general lack of association of water and sanitation variables with cholera risk was surprising given the importance of water in cholera transmission. Rather than a true lack of association, it’s possible that our null results reflect the limitations of using self-reported water and sanitation measures, which may be unreliable.

The proper interpretation of the finding that rural children who received vitamin A were at higher cholera risk than those who did not is unclear, and the criteria by which children received vitamin A supplementation are unknown. Retinol deficiency is more common in children with cholera [24]. If supplementation was based on retinol deficiency, and those with prior supplementation are at continued risk for retinol deficiency, then the observed increased risk for cholera hospitalization among children who received vitamin A supplementation is to be expected. Alternatively, vitamin A deficiency may be a surrogate for malnutrition, which is also known to be associated with cholera severity or duration [25]. However, severe acute malnutrition was not associated with cholera risk in our data.

The increased risk associated with having a family member with diarrhea in the past week has also been found in studies of non-cholera diarrhea [29], [30]. In our study, the increased risk is likely due to shared primary exposures as well as genetic/familial susceptibility [26], [27] and secondary person-to-person transmission through environmental contamination [28].

The use of anonymized data prevented us from assessing repeat visits by the same patient. However, since cholera infection confers natural immunity [31], it is unlikely that an individual would contribute more than one cholera case to our study. This is confirmed by a previous study that found only three repeat cholera hospitalizations out of more than 7,000 cholera cases over a 15-year period [19]. Nonetheless, we cannot rule out the possibility that a patient classified in this study as having non-cholera diarrhea might have had cholera in the past. This possible misclassification might have led to over- or underestimation of associations. We were also unable to assess family clustering of diarrheal cases in the DDSS. Though clustering could lead to violations of underlying independent observation assumptions [26], [32], with a sample this large, any clustering effects are likely to be minimal. In addition, antibiotic use prior to hospitalization, which is known to occur in Bangladesh [33], could not be assessed. This could have skewed the DDSS data, since antibiotic treatment is highly efficacious. Despite these limitations, the large sample size, well-defined population, systematic sampling, and expert laboratory diagnosis of *V. cholerae* are strengths of this study, as is the fact that our referent group is comprised of hospital patients with other causes of diarrhea. Our study therefore highlights risk factors unique to cholera, as opposed to general diarrheal risks [34], [35].

In conclusion, we report that increasing age, measures of SES, maternal education, and current breastfeeding status are key correlates of risk for cholera hospitalization among children under five in rural and urban Bangladesh. In addition, a history of vitamin A supplementation and having a family member with diarrhea in the past week were associated with increased risk among rural children. The lack of association with water and sanitation measures highlights the need for a more thorough assessment of potential waterborne exposures. Continued attention should be directed to promotion of breastfeeding, female education, securing viable livelihoods, and promulgation of safe water sources. Finally, the risk faced by family members of cholera cases may warrant renewed research regarding the use of targeted chemoprophylaxis in endemic rural settings [33], [36].
